# Light Emitting Diode Photobiomodulation Enhances Oxidative Redox Capacity in Murine Macrophages Stimulated with *Bothrops jararacussu* Venom and Isolated PLA_2_s

**DOI:** 10.1155/2022/5266211

**Published:** 2022-07-15

**Authors:** Valdison Pereira dos Reis, Sulamita da Silva Setúbal, Alex A. Ferreira e Ferreira, Hallison Mota Santana, Milena Daniela Souza Silva, Ortência De Oliveira Sousa, Charles Nunes Boeno, Andreimar M. Soares, Stella R. Zamuner, Juliana P. Zuliani

**Affiliations:** ^1^Laboratório de Imunologia Celular Aplicada à Saúde, FIOCRUZ Rondônia, Porto Velho, RO, Brazil; ^2^Laboratório de Biotecnologia de Proteínas e Compostos Bioativos da Amazônia Ocidental, Centro de Estudos de Biomoléculas Aplicadas à Saúde (CEBio)–Fundação Oswaldo Cruz, FIOCRUZ Rondônia, Porto Velho, Rondônia, Brazil; ^3^Laboratório de Análise e Visualização de Dados-Fundação Oswaldo Cruz, FIOCRUZ Rondônia, Porto Velho, Rondônia, Brazil; ^4^Universidade Nove de Julho (UNINOVE), São Paulo, SP, Brazil

## Abstract

Photobiomodulation therapy associated with conventional antivenom treatment has been shown to be effective in reducing the local effects caused by bothropic venoms in preclinical studies. In this study, we analyzed the influence of photobiomodulation using light emitting diode (LED) on the oxidative stress produced by murine macrophages stimulated with *Bothrops jararacussu* venom and it isolated toxins BthTX-I and BthTX-II. Under LED treatment, we evaluated the activity of the antioxidant enzymes catalase, superoxide dismutase, and peroxidase as well as the release of hydrogen peroxide and the enzyme lactate dehydrogenase. To investigate whether NADPH oxidase complex activation and mitochondrial pathways could contribute to hydrogen peroxide production by macrophages, we tested the effect of two selective inhibitors, apocynin and CCCP3, respectively. Our results showed that LED therapy was able to decrease the production of hydrogen peroxide and the liberation of lactate dehydrogenase, indicating less cell damage. In addition, the antioxidant enzymes catalase, superoxide dismutase, and peroxidase increased in response to LED treatment. The effect of LED treatment on macrophages was inhibited by CCCP3, but not by apocynin. These findings show that LED photobiomodulation treatment protects macrophages, at least in part, by reducing oxidative stress caused *B. jararacussu* venom and toxins.

## 1. Introduction

Snakebite envenoming is an important public health neglected tropical disease, especially in low- and middle-income countries such as Brazil [1]. Snakebites can cause serious systemic effects and locally dangerous signs and symptoms including bleeding, swelling, pain, and hemorrhage. The primary treatment for snakebite envenoming is administering the antivenom serum therapy medicine that binds to and inactivates snake venom toxins, helping to prevent negative outcomes. Serum therapy is a standard treatment for snake envenoming effective against systemic damage, but it has limitations for the treatment of local tissue damage, often resulting in permanent motor disability, a frequent problem observed for these types of accidents [2].

As standard serum therapy has limitations to neutralize locally the snake toxins, many efforts are under investigation associating this medicine to animals' serum inhibitors, molecules isolated from plants, camelid nanobodies, and recently photobiomodulation therapy to reduce the local effects induced by snake toxins. Photobiomodulation therapy with *light emitting diode* (LED) is a noninvasive form of phototherapy that utilizes wavelengths of light between 600 and 1000 nm to deliver low irradiance and doses to the target tissue [3] widely used in several areas of medicine due to its beneficial local effects such as analgesics, anti-inflammatory, and healing effects [4, 5]. It promotes biochemical changes within cells where photons are absorbed by photoreceptors, causing chemical changes that promote cell survival and differentiation, muscle regeneration, tissue fibrosis prevention, and inflammatory process modulation [6, 7].

The myotoxicity caused by snake venoms induces an influx of ions and the release of proteins into the extracellular environment, promoting the necrosis of muscle fibers and resulting in an acute inflammatory response [8–10]. In envenoming experimental animal model studies, LED photobiomodulation reduced the venom-induced local effects such as myonecrosis, edema, pain, and hemorrhage [11–13] reinforcing the benefit of this phototherapy.

Pereira dos Reis et al. [14], Reis et al. [15], and Reis et al. [16] showed that LED photobiomodulation also has beneficial properties *in vitro*. When the phototherapy was applied to the elicited macrophages under snake venom or toxins effects, they were protected from death. In addition, it was observed that LED photobiomodulation decreased reactive oxygen species and nitric oxide but increased both phagocytosis and lipid droplets formation.

Therefore, the aim of this study is to analyze the LED photobiomodulation effect on the oxidative stress in elicited macrophages under *Bothrops jararacussu* venom and its main toxins BthTX-I and BthTX-II action to understand the role of these radicals in envenoming, since, when produced in large quantities, they can contribute to the local damage in envenoming.

## 2. Materials and Methods

### 2.1. Chemicals and Reagents

RPMI-1640, Hank's solution, L-glutamine, gentamicin, phorbol 12-myristate 13-acetate (PMA), phenol red, Apocynin, and CCCP3 were purchased from Sigma-Aldrich Chem. (MO, USA) (MFCD00001848). Fetal bovine serum was obtained from Cultilab (MG, Brazil). LDH quantification assay kit was obtained from Labtest (Minas Gerais, Brasil). Catalase (ab83464), superoxide dismutase (SOD) (ab65354), and peroxidase (ab155895) assay kits were purchased from ABCAM (Cambridge, UK). All salts and reagents used were obtained from Merck (Darmstadt, Germany) with low endotoxin or endotoxin-free grades.

### 2.2. Venom and Toxins

*Bothrops jararacussu* snake venom (BjV) dehydrated and stored at a temperature of −20°C was acquired from the Amazon Venom Bank at CEBIO-UNIR, RO. Bothropstoxins: BthTX-I, a Lys-49 PLA_2_-like, and BthTX-II, an Asp-49 PLA_2_, were isolated from *B. jararacussu* snake venom at Centro de Estudos de Biomoléculas Aplicadas à Saúde – CEBio (IBAMA, no 27131-2 and CGEN no 010627/2011-1 licenses). The proteins were purified using the CM-Sepharose column and the resulting fractions were subjected to a second phase chromatographic process using C18 reversed-phase column, as described by Andrião-Escarso et al. [17]. Bothropstoxins were dehydrated and stored at a temperature of 4°C and acquired from the Amazon Venom Bank at CEBIO-UNIR-RO.

### 2.3. Endotoxin Quantification

For this assay, the Pierce™ Chromogenic Endotoxin Quant Kit was used. The plate was prewarmed to 37°C, followed by the addition of 50 *μ*L of BthTX-I, BthTX-II, and standards. Then, 50 *μ*L was added and incubated at 37°C for 30 min. After this period, 100 *μ*L of the chromogenic substrate was added and incubated at 37°C for 6 min. After that, the stopping solution was added. Absorbance was measured spectrophotometrically (Bio-Tek Synergy HT Multi-Detection, Winooski, VT) at 405 nm.

### 2.4. LED (*Light Emitting Diode*) Treatment

Cells were irradiated with a 945 nm infrared LED device (model Super Bright LEDs, Inc., St. Louis, MO, USA) immediately upon addition of the venom or toxin in the culture and LED was applied with direct contact to the bottom of the well plate in a continuous mode for 30 s [14–16]. The experiments were conducted in an environment with partial obscurity to not suffer interference from external light. The output power of the LED equipment was measured using the Laser Check power meter (MM Optics, São Carlos, Brazil). The LED parameters, low enough to avoid any thermal effect, were chosen based on previous studies [11, 14–16]. The experimental parameters for the LED are presented in Table 1.

### 2.5. Animals

Swiss male mice weighing 18-20 g were used for the assays. These animals were housed in temperature-controlled rooms and received water and food *ad libitum* until used which is in accordance with the guidelines of the Brazilian College for Animal Experimentation (COBEA) approved by the Committee for Ethics in Animals Utilization Committees of FIOCRUZ-RO protocol number 2019/19.

### 2.6. Harvesting of Macrophages

Thioglycollate-elicited macrophages (TG-macrophages) were harvested 96 h after an intraperitoneal (i.p.) injection of 1 mL of 3% thioglycollate. In brief, animals were killed under halothane and exsanguinated. Then, peritoneal lavage was performed, after a gentle massage of the abdominal wall, using 3 mL of cold phosphate-buffered saline (PBS: 14 mM NaCl, 2 mM NaH_2_PO_4_H_2_O, 7 mM Na_2_HPO_4_12H_2_O) pH 7.2. The peritoneal fluid, containing TG-macrophages, was collected. Total peritoneal cell counts were determined in Neubauer's chamber. The cell population consisted of more than 95% TG-macrophages, as determined by morphological criteria [15, 16, 18–20].

### 2.7. Superoxide Dismutase (SOD), Catalase, and Peroxidase Enzymes Determinations

TG-macrophages (2x10^5^) obtained according to item 2.6 were incubated with RPMI (negative control), BjV (25 *μ*g/mL), and BthTX-I or BthTX-II (25 *μ*g/mL) for 90 min, at 37°C in a humid atmosphere (5% CO_2_) in the presence or absence of LED photobiomodulation. After this period, the cells were centrifuged for 1100 x*g* for 15 min at 4°C and the SOD, catalase, and peroxidase enzymatic activities were determined by commercial kits from Abcam (Cambridge, UK). Absorbances and fluorescences were determined in BioTek Synergy HT MultiDetection (Winooski, VT) at 450 nm and excitation/emission 535/587 nm and expressed in *μ*m/mL or Pm/mL [21, 22].

### 2.8. Cytotoxic Assays

#### 2.8.1. Cell Viability under Apocynin and CCCP3 Pharmacological Treatments

Mitochondrial activity was measured to assess cell viability according to Pereira dos Reis et al. [16]. To verify the toxicity of apocynin (NADPH oxidase inhibitor) and CCCP3 (mitochondrial ROS inhibitor) [23–25], TG-macrophages (2x10^6^ cells/mL) were suspended in an RPMI supplemented with gentamicin (100 *μ*g/mL), L-glutamine (2 mM), and 10% fetal bovine serum. Then TG-macrophages (2x10^5^ cells/100 *μ*L) were incubated in triplicate in 96-well plates with RPMI (control), apocynin (350 nM), and CCCP3 (240 nM) for 90 min, at 37°C in a humid atmosphere with 5% CO_2_ with or without LED photobiomodulation. Next, 10 *μ*L of MTT (5 mg/mL) was added and incubated for 2 h. After centrifugation at 400 xg for 5 min, the supernatant was removed and 100 *μ*L of DMSO was added to dissolve the formed crystals. Subsequently, the plates were kept for 15 min at room temperature and evaluated in a spectrophotometer at 540 nm. The results showed that both inhibitors were not toxic to TG-macrophages with or without LED photobiomodulation (nonirradiated) (Supplementary Figure 1).

#### 2.8.2. Cell Integrity under Apocynin and CCCP3 Pharmacological Treatments

To verify the damage to the cell's plasma membrane, the LDH released into the cell culture supernatant was measured. In brief, TG-macrophages (2x10^5^ cells/mL) were dispensed in 96-well plates and incubated with RPMI (control), apocynin (350 nM), and CCCP3 (240 nM) for 30 min, at 37°C in a humid atmosphere with 5% CO_2_. Subsequently, TG-macrophages were stimulated with RPMI (control), BjV (25 *μ*g/mL), and BthTX-I or BthTX-II (25 *μ*g/mL) for 90 min, at 37°C in a humid atmosphere (5% CO_2_) in the presence or absence of LED photobiomodulation. LDH was quantified in the supernatant of these incubations using the LDH Liquiform kit according to the manufacturer's instructions. Initial absorbance was registered after 1 min and the second register after 2 min from the first one. Absorbances were conducted in BioTek Synergy HT MultiDetection (Winooski, VT) with 340 nm. Results were expressed in U/L, according to Pereira dos Reis et al. [16] and Silva et al. [26].

### 2.9. Hydrogen Peroxide Production Assay

TG-macrophages (2x10^5^) obtained according to item 2.6 were incubated with Hank's solution (1.26 mM CaCl_2_; 5.33 mM KCl; 0.44 mM KH_2_PO_4_; 0.50 mM MgCI_2_; 0.41 mM MgSO_4_; 138 mM NaCl; 4.0 nM NaHCO_3_; 0.30 Mm Na_2_HPO_4_; 5.60 Mm C_6_H_12_O_6_; and 0.56 mM phenol red) (negative control) and apocynin (350 nM) and CCCP3 (240 nM) for 30 min, at 37°C in a humid atmosphere with 5% CO_2_. After this period, TG-macrophages were submitted to incubation with PMA (positive control), BjV (25 *μ*g/mL), and BthTX-I or BthTX-II (25 *μ*g/mL) for 90 min, at 37°C in a humid atmosphere (5% CO_2_) in the presence or absence of LED photobiomodulation. Finally, 10 *μ*L of stop solution (NaOH) was added to stop the reaction. Absorbances in BioTek Synergy HT MultiDetection (Winooski, VT) at 620 nm were recorded and concentrations of H_2_O_2_ were estimated from a known standard curve of peroxide in *μ*M [27, 28].

### 2.10. Statistical Analyses

Means and S.E.M. of all data were obtained and compared using two-way ANOVA, followed by a Tukey test with significance probability levels of less than 0.05.

## 3. Results

### 3.1. Effect of LED Photobiomodulation on Superoxide Dismutase (SOD), Catalase (CAT), and Peroxidase Activity from TG-Macrophages Stimulated with BjV, BthTX-I, or BthTX-II

**Figure 1:**
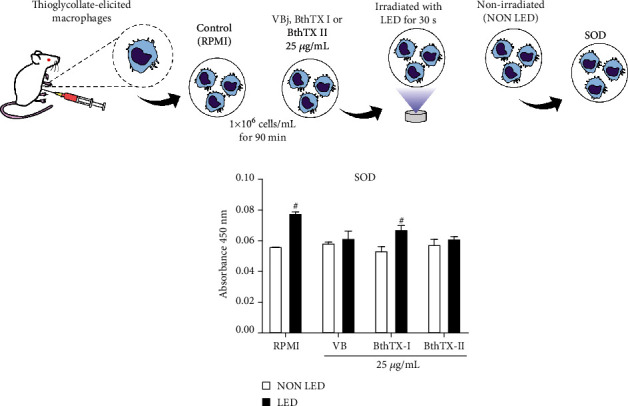
LED photobiomodulation effect on SOD activity by TG-macrophages under *Bothrops jararacussu* snake venom and bothropstoxins stimulation. Thioglycollate-elicited murine macrophages were isolated from peritoneal cavity after 96 h of thioglycollate injection. 2x10^5^ cells were incubated for 90 min with RPMI (control), *Bothrops jararacussu* venom, or bothropstoxins (25 *μ*g/mL) under LED irradiation or without (non LED) at 37°C in a humid atmosphere of 5% CO_2_. SOD activity was quantified by spectrophotometric absorbance measurement at 450 nm. The results were expressed in absorbance and represent the mean ± S.E.M. of 5 animals. ∗*p* < 0.05 in comparison to control group (RPMI) and #*p* < 0.05 in comparison to the respective control group (ANOVA).

Superoxide dismutase (SOD) is an antioxidant enzyme that protects organisms from oxidative stress by catalytically converting O_2_ to hydrogen peroxide (H_2_O_2_) [29]. BjV and both toxins, BthTX-I and BthTX-II, induce ROS production, and LED photobiomodulation reduces this effect [14, 15]. According to the results obtained at the time interval studied, TG-macrophages stimulated with BjV or bothropstoxins did not alter the SOD activity. When the LED photobiomodulation was applied, it was observed an increase in SOD activity in the groups stimulated with RPMI and BthTX-I (Figure 1).

**Figure 2:**
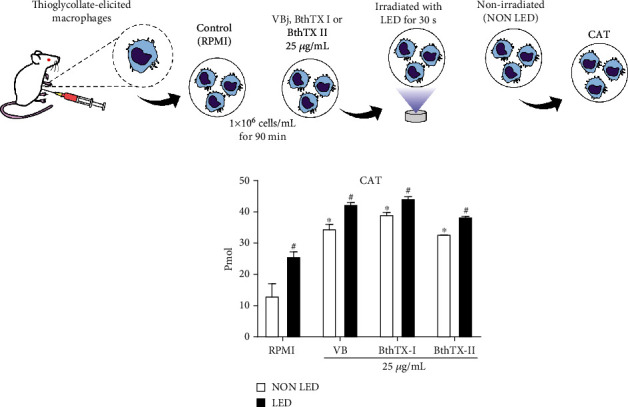
LED photobiomodulation effect on CAT activity by TG-macrophages under *Bothrops jararacussu* snake venom and bothropstoxins stimulation. Thioglycollate-elicited murine macrophages were isolated from peritoneal cavity after 96 h of thioglycollate injection. 2x10^5^ cells were incubated for 90 min with RPMI (control), *Bothrops jararacussu* venom, or bothropstoxins (25 *μ*g/mL) under LED irradiation or without (non LED) at 37°C in a humid atmosphere of 5% CO_2_. CAT activity was quantified by spectrophotometric measurement of the fluorescence at excitation/emission 535/587 nm. The results were expressed in p mol/mL and represent the mean ± S.E.M. of 5 animals. ∗*p* < 0.05 in comparison to control group (RPMI) and #*p* < 0.05 in comparison to the respective control group (ANOVA).

The next enzyme studied was catalase (CAT), an enzyme that prevents cell oxidative damage by degrading hydrogen peroxide to water and oxygen (2H_2_O_2_ ➔ 2 H_2_O + O_2_). The results obtained showed that BjV, as well as BthTX-I and BthTX-II, induced a significant activity of CAT. When the LED photobiomodulation was applied, CAT activity increased in a statistically significant way for all the groups studied (Figure 2).

**Figure 3:**
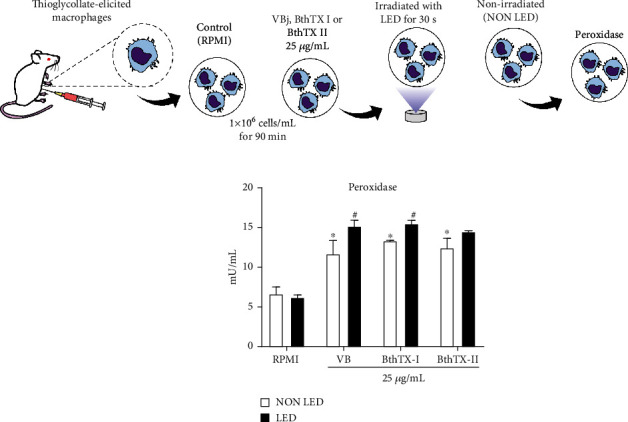
LED photobiomodulation effect on peroxidase activity by TG-macrophages under *Bothrops jararacussu* snake venom and bothropstoxins stimulation. Thioglycollate-elicited murine macrophages were isolated from peritoneal cavity after 96 h of thioglycollate injection. 2x10^5^ cells were incubated for 90 min with RPMI (control), *Bothrops jararacussu* venom, or bothropstoxins (25 *μ*g/mL) under LED irradiation or without (non LED) at 37°C in a humid atmosphere of 5% CO_2_. Peroxidase activity was quantified by spectrophotometric fluorescence measurement at excitation/emission 535/587 nm. The results were expressed in mU/mL of peroxidase activity and represent the mean ± S.E.M. of 5 animals. ∗*p* < 0.05 in comparison to control group (RPMI) and #*p* < 0.05 in comparison to the respective control group (ANOVA).

Peroxidase is an enzyme that breaks down hydrogen peroxide (H_2_O_2_) in a mechanism similar to CAT. In Figure 3, BjV, BthTX-I, and BthTX-II induced a significant activity of peroxidase but LED photobiomodulation induced a significant increase of peroxidase activity in the groups stimulated with BjV and BthTX-I.

### 3.2. Effect of LED Photobiomodulation on Hydrogen Peroxide Liberation and LDH Release from TG-Macrophages Stimulated with BjV, BthTX-I, or BthTX-II

Pereira dos Reis et al. [14] and Reis et al. [15] showed that BjV as well as BthTX-I and BthTX-II induced ROS production and LED photobiomodulation was effective in decreasing this effect. Here, the hydrogen peroxide production was investigated under BjV as well as BthTX-I and BthTX-II stimulation.

Firstly, the cytotoxic effect of the inhibitors used for hydrogen peroxide determination was analyzed using an MTT assay. Both inhibitors at the concentrations used did not show cytotoxic effect for TG-macrophages (Supplementary Figure 1).

**Figure 4:**
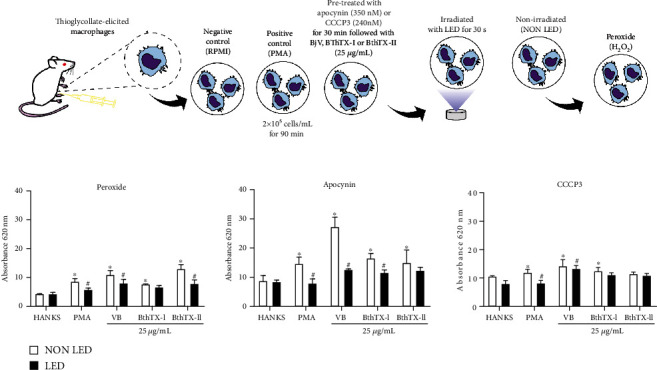
LED photobiomodulation effect on hydrogen peroxide liberation by TG-macrophages under *Bothrops jararacussu* snake venom and bothropstoxins stimulation. Thioglycollate-elicited murine macrophages were isolated from peritoneal cavity after 96 h of thioglycollate injection. 2x10^5^ cells were incubated for 30 min with the inhibitors apocynin (350 nM) or CCCP 3 (240 nM) followed by 90 min of stimulation of cells with Hanks (control), PMA (500 ng/mL; positive control), *Bothrops jararacussu* venom, or bothropstoxins (25 *μ*g/mL) under LED irradiation or without (non LED) at 37°C in a humid atmosphere of 5% CO_2_. Hydrogen peroxide liberation was quantified by spectrophotometric absorbance measurement at 620 nm. The results were expressed in absorbance and represent the mean ± S.E.M. of 5 animals. ∗*p* < 0.05 in comparison to control group (Hanks) and #*p* < 0.05 in comparison to the respective control group (ANOVA).

Then, using Apocynin (350 nM) to inhibit the NADPH oxidase pathway, and CCCP3 (240 nM) to inhibit the mitochondrial pathway, the hydrogen peroxide production by TG-macrophages under LED photobiomodulation was investigated. In Figure 4(a), it is possible to observe that TG-macrophages stimulated with PMA as well as BjV or both toxins, BthTX-I and BthTX-II, induced the production of hydrogen peroxide. When LED photobiomodulation has applied this effect was reduced except for BthTX-I. When the TG-macrophages were pre-treated with Apocynin, it was not observed a decrease in H_2_O_2_ production, but an increase that was reduced in the presence of LED photobiomodulation (Figure 4(b)). The data with CCCP3, another inhibitor used but for mitochondrial pathway, showed that this inhibitor although does not inhibit the formation of hydrogen peroxide under BjV and both bothropstoxins in the presence of LED photobiomodulation this effect was reduced (Figure 4(c)).

**Figure 5:**
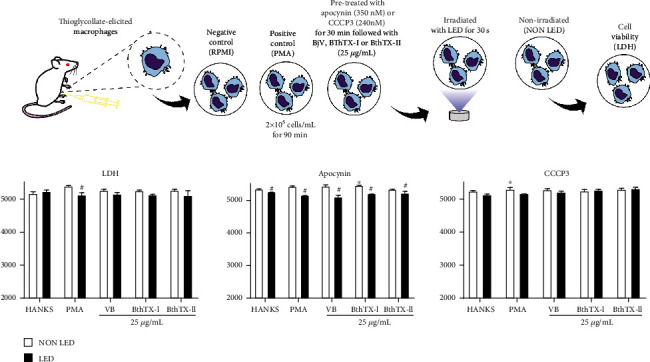
LED photobiomodulation effect on LDH liberation by TG-macrophages under *Bothrops jararacussu* snake venom and bothropstoxins stimulation. Thioglycollate-elicited murine macrophages were isolated from peritoneal cavity after 96 h of thioglycollate injection. 2x10^5^ cells were incubated for 30 min with the inhibitors apocynin (350 nM) or CCCP 3 (240 nM) followed by 90 min of stimulation of cells with Hanks (control), PMA (500 ng/mL; positive control), *Bothrops jararacussu* venom, or bothropstoxins (25 *μ*g/mL) under LED irradiation or without (non LED) at 37°C in a humid atmosphere of 5% CO_2_. LDH liberation was quantified by spectrophotometric absorbance measurement at 340 nm. The results were expressed in absorbance and represent the mean ± S.E.M. of 5 animals. ∗*p* < 0.05 in comparison to control group (RPMI) and #*p* < 0.05 in comparison to the respective control group (ANOVA).

The supernatants of these incubations were collected for lactate dehydrogenase (LDH) release determination as a parameter of membrane integrity and cell death. Thus, it is possible to observe that in all groups, it was found LDH release that was not different from controls (Figure 5(a)–5(c)). However, LED photobiomodulation decreased the LDH release in the group treated to apocynin and simulated with BjV or both toxins, BthTX-I and BthTX-II (Figure 5(b)).

## 4. Discussion

The literature contains few studies on the use of photobiomodulation to treat the local effects induced by bothropic venoms in which they demonstrate the results of this therapy in different contexts, with various types of analysis, but always demonstrating its effectiveness as a supporting treatment in snakebite envenomation. In the present study, we evaluated the effect of LED photobiomodulation on redox oxidative capacity produced by murine macrophages stimulated with *Bothrops jararacussu* venom and it isolated toxins BthTX-I and BthTX-II.

Although the mechanism of action of LED is not fully understood, the literature suggests that its effects are based on the emission of photons generated by the device, which, upon entering cells, are immediately absorbed by intracellular chromophores in mitochondria, known as cytochrome c oxidase. This absorption is converted into metabolic energy-producing adenosine triphosphate (ATP), consequently increasing the metabolism of that cell, leading to an increase in cellular functions, as well as an increase in the synthesis of RNA and DNA [6, 30, 31] thus promoting the increased cell viability [14, 15], among other benefits.

In the context of snakebite envenomation, these free radicals are unstable molecules that, in aggravated conditions, might confer cytotoxicity [32], which may intensify the harmful effects caused by the envenomation. Moreover, Pereira dos Reis et al. [14] and Reis et al. [15], in their study, clearly showed that LED photobiomodulation therapy dramatically reduced the generation of these free radicals in thioglycollate-elicited peritoneal macrophages incubated with *Bothrops jararacussu* venom, BthTX-I, and BthTX-II. These findings contrast with some previously published studies that claim that photobiomodulation enhances the generation of these radicals [4, 6, 30, 31].

Antioxidant enzymes like superoxide dismutase (SOD), catalase (CAT), and peroxidase constitute a first-line antioxidant defense system that plays a key and fundamental role in the total defense mechanisms and strategies in biological systems. SOD is a universal enzyme found in all living things that catalyze the reduction of superoxide to oxygen and hydrogen peroxide, thereby regulating the levels of reactive oxygen and nitrogen (NO) in the cell. They are considered the first line of defense against free radicals [33, 34]. Other enzymes, such as CAT and glutathione peroxidase, will destroy the superoxide anion once it has been dismutated into hydrogen peroxide [35–37]. Under normal circumstances, the biological system activates SOD, which specifically maintains the O_2_^•–^ concentration at an optimal level [38]. These antioxidant enzymes, acting together or separately, regulate the cytotoxic process of free radicals in the inflammatory process, especially when they are generated under aggravated conditions.

As a result, our findings revealed a significant role for LED photobiomodulation treatment in the synthesis of these three antioxidant enzymes in murine macrophages, in response to the *Bothrops jararacussu* venom, BthTX-I, and BthTX-II, and the negative control groups [14, 15]. In a previous study from our group, was demonstrated that thioglycollate-elicited peritoneal macrophages treated with LED photobiomodulation produced significantly less ROS and NO under bothropic venom incubation. We believe that the reductions previously seen by our research group are attributed to the influence of LED in raising the synthesis of antioxidant enzymes, such as SOD, CAT, and peroxidase in the irradiated group. As a result, an increase in these enzymes would first dismutate superoxide and then hydrogen peroxide, explaining the reductions in ROS and NO seen in our early experiments.

Although the literature does not present published data directly involving the effect of LED photobiomodulation on redox oxidative reductions in snakebite studies, the data by Silva Macedo et al. [39] demonstrate an important oxidative redox capacity of photobiomodulation. Their findings showed an increase in the synthesis of antioxidant enzymes in lung cells, such as peroxidase, suggesting that photobiomodulation may be a useful tool for the treatment of various lung diseases.

We started quantifying hydrogen peroxide formation, followed by an assay of LDH quantification, in thioglycollate-elicited peritoneal macrophages stimulated with *Bothrops jararacussu* venom, BthTX-I, and BthTX-II submitted or not to LED photobiomodulation. We also evaluated the two possible sources of ROS production, the mitochondrial pathway and the NADPH oxidase complex activation pathway using a pharmacological approach with two inhibitors, the carbonylcyanide m-chlorophenylhydrazone (CCCP3), an uncoupler of the respiratory chain complex III (inhibitor of the mitochondrial pathway), and apocynin, an NADPH oxidase inhibitor. The results showed that the inhibitors did not confer toxicity to the cells at the concentrations used, and the venom and the toxins caused an increase of superoxide anion and LDH, which corroborate with the literature [14, 15, 40].

Herein, the data showed a considerable reduction of hydrogen peroxide after LED photobiomodulation in all groups of cells stimulated with the venom or the toxins. When the inhibitors were used, there was an increase in hydrogen peroxide production by murine macrophages under *Bothrops jararacussu* venom, BthTX-I and BthTX-II stimulation which was reduced with LED photobiomodulation. This effect was not observed for the LDH assay. In the case of hydrogen peroxide, it is possible that when one pathway was inhibited, the cell use the other one to produce the mediator, but when LED photobiomodulation was applied, the hydrogen peroxide production was reduced to basal levels, evidencing the ability of LED to reduce this mediator.

In conclusion, our presented data showed important oxidative redox characteristics of LED and again its contribution to the reduction of free radicals. Furthermore, our studies reinforce the importance of using this therapy in association with conventional treatment with serum therapy, to reduce the local effects caused by snakebite envenomation. This study contributes with other studies in the literature to understand the mechanisms of photobiomodulation, as an important intervention tool to treat local effects, in the context of snakebites.

## Figures and Tables

**Table 1:** Protocol for LED irradiation.

Parameters	LED
Wavelength (mm)	945
Energy density (J/cm^2^)	3
Mean power output (mW)	120
Irradiation time (seg)	30
Beam area (cm^2^)	1.2
Energy per point (J)	3.6

## Data Availability

Data are available on request from the corresponding author.

## Supplementary Materials

Supplementary Materials Supplementary Figure 1 Cytotoxic effect of apocynin and CCCP3 on TG-macrophages. Thioglycollate-elicited murine macrophages were isolated from peritoneal cavity after 96 h of thioglycollate injection. 2x10^5^ cells were incubated for 90 min with RPMI (control), apocynin (350 nM), or CCCP3 (240 nM) at 37°C in a humid atmosphere of 5% CO_2_ under LED irradiation or without (non LED). Cells viability was assessed using the MTT method quantified by spectrophotometric absorbance measurement at 340 nm. The results were expressed in absorbance and represent the mean ± S.E.M. from 4-5 animals. ∗*p* < 0.05 in comparison to control group (RPMI) and #*p* < 0.05 in comparison to the respective non treated group (ANOVA).
