# Supplementary figures and images for: Light Emitting Diode Photobiomodulation Enhances Oxidative Redox Capacity in Murine Macrophages Stimulated with Bothrops jararacussu Venom and Isolated PLA2s
Source: Biomed Res Int. 2022 Jul 15;2022:5266211. doi: 10.1155/2022/5266211 (PMC9307370; doi:10.1155/2022/5266211)

Supplementary Figure 1

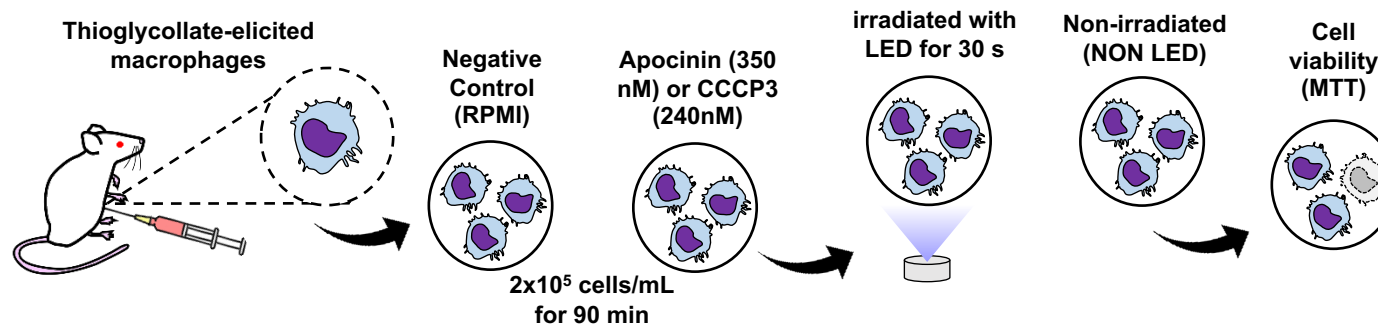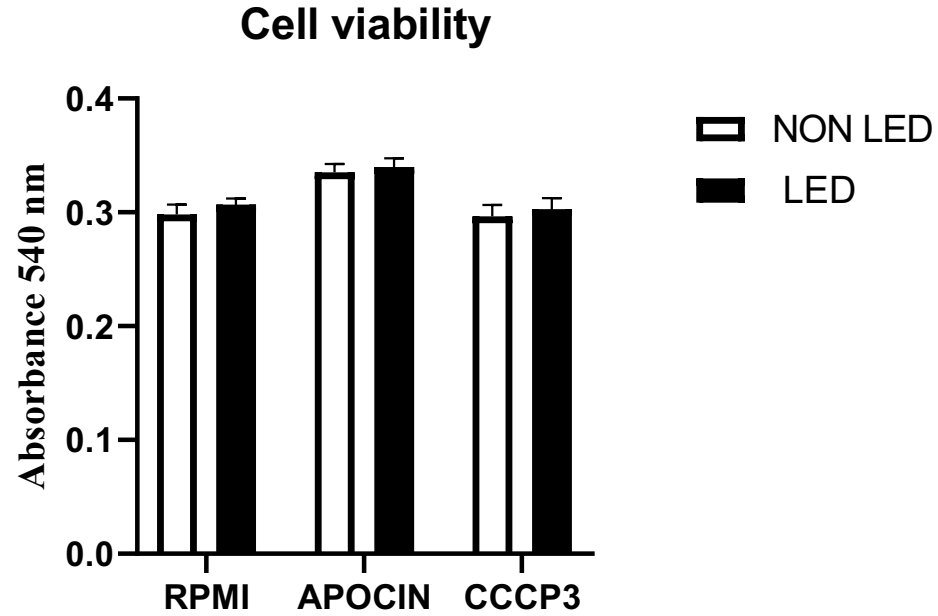

Supplement: Supplementary Materials — Supplementary Figure 1 Cytotoxic effect of apocynin and CCCP3 on TG-macrophages. Thioglycollate-elicited murine macrophages were isolated from peritoneal cavity after 96 h of thioglycollate injection. 2x10^5^ cells were incubated for 90 min with RPMI (control), apocynin (350 nM), or CCCP3 (240 nM) at 37°C in a humid atmosphere of 5% CO2 under LED irradiation or without (non LED). Cells viability was assessed using the MTT method quantified by spectrophotometric absorbance measurement at 340 nm. The results were expressed in absorbance and represent the mean ± S.E.M. from 4-5 animals. ∗p < 0.05 in comparison to control group (RPMI) and #p < 0.05 in comparison to the respective non treated group (ANOVA). [file 5266211.f1.pdf]
